# Erysipelas and Puerperal Fever

**Published:** 1902-06-21

**Authors:** 


### ERYSIPELAS AND PUERPERAL FEVER.

In his Presidential Address before the Hunterian Society Dr. Galabin gave the results of a careful inquiry which he has made in regard to the annual variation of puerperal fever compared with that of certain allied diseases, namely, erysipelas, septicæmia and pyæmia, scarlet fever, and rheumatism. So far as can be seen by plotting out in graphic form the mortality from these several diseases, there appears to be but little relation between the annual prevalence of puerperal fever and that of either septicæmia, scarlet fever, or rheumatism, which is interesting when one remembers for how long a time the contagion of scarlet fever was held to be an important cause of puerperal septicæmia. Not only is there but little similarity between the curves formed by plotting out the annual mortality of scarlet and puerperal fever for 20 years, but the curves formed by the mortality from these diseases, taken week by week, are absolutely different, scarlet fever rising to a maximum at the end of October and falling considerably before puerperal fever rises to its main maximum in early January. By far the most striking result of Dr. Galabin's inquiry is the accurate and precise manner in which the curves for erysipelas and for puerperal fever are shown to correspond, whether they are taken by the year or by the week, or are compiled from the London statistics or from those of the country at large. In the annual curves almost every deviation in the one is represented in the other. A still more remarkable proof of the close relation of erysipelas and puerperal fever is to be found on comparing the curves of weekly variation. "Every curve and turn and every date when the curves cross the mean correspond. . . . This resemblance of the curves confirms the evidence of bacteriology as to the close connection between puerperal fever and erysipelas." The conclusion to be drawn is that the contagion of erysipelas is more dangerous to the puerperal woman than that of any other septic disease other than puerperal fever itself.

